# Socioeconomic Status and Major Adverse Transplant Events in Pediatric Heart Transplant Recipients

**DOI:** 10.1001/jamanetworkopen.2024.37255

**Published:** 2024-10-03

**Authors:** Christina Hartje-Dunn, Kimberlee Gauvreau, Heather Bastardi, Kevin P. Daly, Elizabeth D. Blume, Tajinder P. Singh

**Affiliations:** 1 Department of Cardiology, Boston Children’s Hospital, Boston, Massachusetts; 2 Department of Pediatrics, Harvard Medical School, Boston, Massachusetts; 3 Department of Biostatistics, Harvard School of Public Health, Boston, Massachusetts; 4 Now with Seattle Children’s Hospital, Seattle, Washington

## Abstract

**Importance:**

Low socioeconomic status (SES) has been associated with higher risk of rejection and graft loss in pediatric heart transplant (HT) recipients. The association of SES with other posttransplant morbidities is unknown.

**Objective:**

To assess whether low SES is associated with higher risk of a major adverse transplant event (MATE) among pediatric HT recipients.

**Design, Setting, and Participants:**

Retrospective single-center cohort study at a children’s hospital in Boston with consecutive primary HT recipients from 2006 to 2019 and follow-up through 2022. Data were analyzed from June 2023 to March 2024.

**Exposure:**

Very low or low, moderate, and high or very high Childhood Opportunity Index (COI) for neighborhood (census tract) of patient residence.

**Main Outcomes and Measures:**

Primary outcome was 3-year MATE-6 score assessed in 6-month survivors as cumulative burden of acute cellular rejection, antibody-mediated rejection, coronary vasculopathy, lymphoproliferative disease, kidney dysfunction, and infection, each as an ordinal score from 0 to 4 (24 for death or retransplant). Secondary outcomes were freedom from rejection during first 6 months, freedom from death or retransplant, MATE-3 score for events 1 to 3 (under immune suppression) and events 4 to 6 (chronic immune suppression effects), and each MATE component.

**Results:**

Of 153 children analyzed, the median (IQR) age at HT was 7.2 (1.5-14.8) years, 99 (65%) were male, 16 (10%) were Black, 17 (11%) were Hispanic, and 106 (69%) were White. Fifty patients (33%) lived in very low or low, 17 (11%) in moderate, and 86 (56%) in high or very high COI neighborhoods. There was no significant group difference in mean (SD) 3-year MATE-6 score (very low or low COI, 3.4 [6.5]; moderate COI, 2.4 [6.3]; and high or very high COI, 4.0 [6.9]). Furthermore, there was no group difference in mean (SD) MATE-3 scores for underimmune suppression (very low or low COI, 1.9 [3.5]; moderate COI, 1.2 [3.2]; and high or very high COI, 2.2 [3.6]), chronic immune suppression effects (very low or low COI, 1.6 [3.3]; moderate COI, 1.1 [3.2]; and high or very high COI, 1.8 [3.6]), individual MATE components, rejection during the first 6 months, or death or retransplant.

**Conclusions and relevance:**

In this cohort study of pediatric HT recipients, there was no difference in posttransplant outcomes among recipients stratified by SES, a notable improvement from prior studies. These findings may be explained by state-level health reform, standardized posttransplant care, and early awareness of outcome disparities.

## Key Points

**Question:**

What is the association of socioeconomic status (SES) with posttransplant events in pediatric heart transplant (HT) recipients?

**Findings:**

In this cohort study of 153 HT recipients younger than 21 years from 2006 to 2019 with follow-up until 2022, there was no significant difference in cumulative burden of major posttransplant events or risk of death or retransplant when stratified by residence in very low or low, moderate, and high or very high Childhood Opportunity Index neighborhood.

**Meaning:**

In this study, the lack of association between SES and posttransplant outcomes is a notable improvement from prior studies, and may be explained by state-level health care reform, standardized care, and early awareness of disparities.

## Teaser

This cohort study evaluates the association of socioeconomic status with major adverse transplant events among pediatric heart transplant recipients.

## Introduction

It has long been recognized that socioeconomic status (SES) influences an individual’s health outcomes.^1,2^ Among pediatric heart transplant (HT) recipients, Black children and children with low SES have been shown to be at higher risk of rejection and graft loss; the higher risk in Black children is also mediated in part by social and economic factors.^3,4^ It is unknown whether lower graft survival in HT recipients of low SES is entirely due to their higher rejection risk. In addition to acute rejection episodes, the morbidity in HT recipients comes from adverse effects of immunosuppression (IS), and from coronary allograft vasculopathy (CAV), sometimes referred to as chronic rejection.

The purpose of this study was to assess the association of SES with a broader range of posttransplant outcomes in pediatric HT recipients. We used a composite outcome measure, the Major Adverse Transplant Event (MATE) score at 3 years posttransplant, recently described in 6-month HT survivors as cumulative burden of 6 morbidities: acute cellular rejection, antibody-mediated rejection, CAV, lymphoproliferative disease, kidney dysfunction, and infection, each assessed as an ordinal score based on severity.^5^ Our goal was to understand whether low SES is associated with these posttransplant complications and to obtain guidance for potential targets for future interventions.

## Methods

### Study Settings and Participants

The study included all children younger than 21 years who underwent primary HT at Boston Children’s Hospital from 2006 to 2019. The last date of the study was December 31, 2022, allowing a minimum of 3 years of follow-up for all children in the study. Due to contractual requirements, we excluded patients enrolled in the multicenter Tacrolimus and Everolimus Against Tacrolimus and Mycophenolate in Pediatric Heart Transplantation Using the MATE Score (TEAMMATE trial), a study comparing MATE outcomes between children randomized to 1 of 2 different IS regimens.

### Study Design and Variables

This was a retrospective cohort study. The institutional review board at Boston Children’s Hospital approved the study with a waiver of informed consent because the study was retrospective. This report follows the Strengthening the Reporting of Observational Studies in Epidemiology (STROBE) reporting guideline.

We assessed patient demographics and clinical variables such as cardiac diagnosis, pretransplant clinical status, heart failure severity at transplant, and outcome variables as described subsequently. Home address at the time of HT was used to determine the census tract of patient residence. Census tracts are small geographical areas in the US with an average population of 4000, and are relatively homogeneous with regard to economic status and living conditions of their residents. Race and ethnicity were assessed due to known association of Black race with posttransplant outcomes and with lower SES. Race and ethnicity was recorded as reported in the electronic medical record, and analyzed as Black (non-Hispanic Black), White (non-Hispanic White), Hispanic, or Other. We hypothesized that patients of low SES, assessed using Childhood Opportunity Index (COI) for census tract of patient residence, would be at risk of more adverse transplant events and thus would have higher MATE scores.

COI is a composite score of 29 indicators of childhood opportunity across the domains of education, health, environment, and socioeconomic status used to quantify the distribution of opportunity across US neighborhoods (census tracts).^6,7^ This score ranges from 1 to 100 for a given census tract, has been reported for all US census tracts (first for 2010, and then for 2015), and represents a relative assessment of COI compared with all other census tracts nationally in the US. Several studies have associated COI with health care utilization, illness severity, and adverse medical and postsurgical outcomes, including congenital heart surgical procedures.^8,9^ We used census tract of patient residence at HT to determine the COI 2.0 composite score, quintile, and COI level (stratified as very low or low, moderate, or high or very high). To use the neighborhood COI data closest to HT, we used the 2010 COI in children who received HT through 2012, and the 2015 COI in children who received HT in 2013 or later. The primary variable was COI level of patient residence at HT, analyzed as 3 groups of patients, described previously.

The primary outcome was 3-year MATE-6 score assessed in 6-month survivors. This score is a predictor of death and/or graft loss in pediatric HT recipients on longer-term follow-up, and thus allows studies of smaller sample sizes to have adequate statistical power.^5^ This score was used as an end point in a recently completed, federally funded, multicenter, randomized clinical trial (TEAMMATE trial) of immune suppression in pediatric HT recipients.^10^ Each MATE component is assigned an ordinal score of 0 to 4, with a maximum composite score of 24 assigned for patient death or graft loss (retransplant). Each component MATE score was determined by standardized criteria.^5^ For acute cellular rejection (ACR), International Society for Heart and Lung Transplantation (ISHLT) 2010 grading criteria for grade 0 to grade 3R, hemodynamics, and treatment decision were used where grade 0 corresponds to a MATE score of 0 (no disease), and grade 3R or hemodynamic compromise irrespective of grade corresponds to a MATE score of 3 (severe). Similarly, antibody mediated rejection (AMR) was defined by ISHLT pathologic diagnostic criteria^11^ and CAV using the ISHLT grading system.^12^ Lymphoproliferative disease was scored based on morphology (monomorphic vs polymorphic), the number of sites involved, and the presence of high-risk features such as bone marrow or CNS involvement. Kidney function was assessed as estimated creatinine clearance by the modified Schwartz formula,^13^ and MATE score determined based on the chronic kidney disease stage. Finally, MATE score for infection was determined by the duration and route of administration of antibiotic or antiviral agent, infection with specific high-risk organisms, and the clinical severity of infection. Secondary outcomes were percentage of patients with rejection (grade 2R ACR or AMR) during the first 6 months post-HT,^14^ freedom from death or retransplant during the study period, MATE-3 score for events associated with under IS (ACR, AMR, and CAV), MATE-3 score for events related to IS therapy (infection, kidney dysfunction, and lymphoproliferative disease), and individual MATE components.

### Statistical Analysis

The study cohort is described using median (IQR) or mean (SD) for continuous variables, and number (percentage) for categorical variables. Baseline patient characteristics were compared across the 3 levels of COI (very low or low, moderate, and high or very high) using the Kruskal-Wallis test for continuous and ordinal variables, and Fisher exact test for binary and nominal variables. The proportion of patients who experienced acute rejection during the first 6 months post-HT were compared among groups using Fisher exact test. Mean 3-year MATE-6 scores were compared among COI groups using 1-way analysis of variance. Secondary outcomes of MATE components were compared between COI categories using the Kruskal-Wallis test. Time from transplant to death or retransplant was estimated using the Kaplan-Meier method, and compared between groups using the log-rank test. Data were analyzed using Stata version 17 (StataCorp) from June 2023 to March 2024

## Results

### Study Population

Between 2006 and 2019, 170 children younger than 21 years underwent primary HT at Boston Children’s Hospital. Twelve of these were enrolled in the TEAMMATE trial, 4 did not have primary residence in the US, and 1 was transferred to a different institution early post-HT; these children were excluded. The remaining 153 children composed the study cohort. Their median (IQR) age at transplant was 7.2 (1.5-14.8) years, 99 (65%) were male, 16 (10%) were Black, 17 (11%) were Hispanic. and 106 (69%) were White. The indication for HT was cardiomyopathy in 73 (48%), and congenital heart disease in 75 (49%).

The median (IQR) neighborhood COI score was 68 (34-84). Fifty patients (33%) lived in very low or low, 17 (11%) in moderate, and 86 (56%) in high or very high COI neighborhoods. There was a significant difference between the COI groups in the percentage of non-White patients: 52% of patients in the very-low or low COI, 18% of patients in the moderate COI, and 16% of patients in high or very-high COI neighborhoods were non-White (*P* < .001, Fisher exact test). There was no significant difference in pretransplant sensitization or heart failure severity as indicated by UNOS listing status, respiratory support, inotrope use, mechanical support, serum bilirubin, or kidney function at the time of HT among COI groups (Table 1).

**Table 1.** Baseline Characteristics Overall and by Child Opportunity Index Level

Characteristic	Participants, No. (%)				*P* value
	Total cohort (N = 153)	Child Opportunity Index Level			
		Very low/low (n = 50)	Moderate (n = 17)	High/very high (n = 86)	
Age at transplant, median (IQR), y	7.2 (1.5-14.8)	9.5 (1.5-15.0)	1.8 (0.8-5.6)	6.9 (1.9-15.4)	.04
Sex					
Female	54 (35)	18 (36)	3 (18)	33 (38)	.27
Male	99 (65)	32 (64)	14 (82)	53 (62)	
Race and ethnicity					
Black	16 (10)	9 (18)	1 (6)	6 (7)	<.001
Hispanic	17 (11)	14 (28)	1 (6)	2 (2)	
Other	10 (7)	3 (6)	1 (6)	6 (7)	
White	106 (69)	22 (44)	12 (71)	72 (84)	
Declined	4 (3)	2 (4)	2 (12)	0	
Diagnosis					
CMP	73 (48)	28 (56)	7 (41)	38 (44)	.31
CHD	75 (49)	19 (38)	10 (59)	46 (53)	
Other	5 (3)	3 (6)	0	2 (2)	
Single ventricle CHD	51 (33)	14 (28)	7 (41)	30 (35)	.54
Height, median (IQR), cm	114 (71-159)	127 (76-158)	71 (60-102)	115 (75-161)	.07
Weight, median (IQR), kg	20.0 (8.3-49.9)	28.5 (9.3-48.9)	10.0 (6.6-16.8)	20.4 (8.3-55.1)	.09
Body mass index, median (IQR)^a^	16.7 (15.2-19.9)	16.8 (15.4-19.8)	15.5 (15.2-19.6)	16.8 (14.8-20.1)	.71
Pretransplant inotropes	83 (54)	26 (52)	9 (53)	48 (56)	.92
Mechanical support (VAD/ECMO)	50 (33)	17 (34)	6 (35)	27 (31)	.91
Ventilator at transplant	12 (8)	3 (6)	2 (12)	7 (8)	.68
Sensitization PRA ≥10%	55 (36)	21 (42)	4 (24)	30 (35)	.39
Crossmatch positive	17 (11)	7 (14)	1 (6)	9 (10)	.75
UNOS listing status					
1A	122 (80)	38 (76)	12 (71)	72 (84)	.21
1B	14 (9)	5 (10)	4 (24)	5 (6)	
2	17 (11)	7 (14)	1 (6)	9 (10)	
Serum bilirubin, median (IQR), mg/dL	0.4 (0.3-0.8)	0.5 (0.2-0.7)	0.5 (0.3-0.9)	0.4 (0.3-0.8)	.85
Serum creatinine at transplant, median (IQR), mg/dL	0.4 (0.3-0.6)	0.5 (0.3-0.7)	0.3 (0.3-0.6)	0.5 (0.3-0.7)	.87
Creatinine clearance at transplant, median (IQR), mL/min/1.73 m^2^	101 (82-136)	104 (82-135)	92 (74-105)	101 (82-138)	.40
CMV donor/recipient					
Negative/negative	58 (38)	16 (32)	6 (35)	36 (42)	.03
Negative/positive	16 (11)	9 (18)	2 (12)	5 (6)	
Positive/negative	62 (41)	17 (34)	7 (41)	38 (45)	
Positive/positive	15 (10)	8 (16)	1 (6)	6 (7)	
Not documented	1 (1)	0	1 (6)	0	
Ischemic time, median (IQR), min	214 (181-251)	205 (171-240)	214 (186-245)	218 (182-258)	.40

Abbreviations: CHD, congenital heart disease; CMP, cardiomyopathy; CMV, cytomegalovirus; ECMO, extracorporeal membrane oxygenation; PRA, panel reactive antibody; UNOS, united network for organ sharing; VAD, ventricular assist device.

SI Conversion Factors: To convert bilirubin to μmol/L, multiply by 17.104; creatinine to μmol/L, multiply by 88.4; creatinine clearance to mL/s/m^2^, multiply by 0.0167.

^a^ Calculated as weight in kilograms divided by height in meters squared.

### Comparison of MATE Scores Among COI Groups

**Figure 1.**
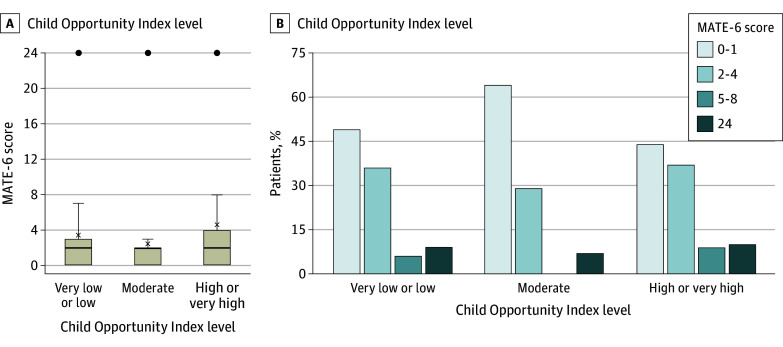
Distribution of Major Adverse Transplant Event (MATE-6) Scores in Pediatric Heart Transplant Recipients Stratified by Childhood Opportunity Index Level A, Each box represents the distribution of MATE-6 scores in the specified Childhood Opportunity Index level. Horizontal lines within the boxes indicate the median score, X represents the mean score, and the bottom and top of each box represent the 25th and 75th percentiles, respectively. The whiskers indicate the smallest and the largest value within 1.5 times the IQR; circles indicate outliers larger than this threshold. B, Illustrates percentage of patients with specific MATE-6 scores.

The mean (SD) MATE-6 score in 142 patients who survived the first 6 months posttransplant was 3.6 (6.7) (Table 2). This compares with a mean (SD) MATE-6 score of 10.0 (4.0) in the cohort where the score was first described (*P* < .001, Fisher exact test).^5^ The median (IQR) MATE-6 score for the cohort was 2 (0-3), with the distribution of MATE-6 scores among the COI groups as shown in Figure 1. There was no difference between the COI groups in mean (SD) MATE-6 scores (3.4 [6.5] in the very low or low COI group, 2.4 [6.3] in the moderate group, and 4.0 [6.9] in the high or very high COI group). Furthermore, there was no difference in mean (SD) MATE-3 scores for under immune suppression (1.9 [3.5] in low or very low, 1.2 [3.2] in moderate, and 2.2 [3.6] in high or very high COI groups), or for immune suppression effects (1.6 [3.3] in very low or low, 1.1 [3.2] in moderate, and 1.8 [3.6] in high or very high COI group). Finally, there was no significant difference between the COI groups in MATE score for any of its 6 components (Table 3).

**Table 2.** Three-Year Major Adverse Transplant Event (MATE) Scores, Overall and by Child Opportunity Index Level^a^

	Total cohort (N = 142)	Child Opportunity Index Level			*P* value
		Very low/low (n = 47)	Moderate (n = 14)	High/very high (n = 81)	
MATE-6					
Mean (SD)	3.6 (6.7)	3.4 (6.5)	2.4 (6.3)	4.0 (6.9)	.68
Median (IQR)	2 (0-3)	2 (0-3)	0 (0-2)	2 (0-4)	.14
MATE-3 at 3 y: immune suppression effects					
Mean (SD)	1.7 (3.4)	1.6 (3.3)	1.1 (3.2)	1.8 (3.6)	.78
Median (IQR)	0 (0-2)	0 (0-2)	0 (0-0)	0 (0-2)	.46
MATE-3 at 3 y: underimmune suppression					
Mean (SD)	2.0 (3.5)	1.9 (3.5)	1.2 (3.2)	2.2 (3.6)	.61
Median (IQR)	0 (0-2)	0 (0-2)	0 (0-0)	0 (0-2)	.16

^a^ The 95% CIs for differences in mean MATE-6 between Childhood Opportunity Index level groups are −2.9 to 5.0 for very low or low vs moderate, and −3.0 to 1.9 for very low or low vs high or very high.

**Table 3.** Components of the Major Adverse Transplant Event (MATE) Score Overall and by Child Opportunity Index Level

MATE	Median (IQR)				*P* value
	Total cohort (N = 142)	Child Opportunity Index Level			
		Very low/low (n = 47)	Moderate (n = 14)	High/very high (n = 81)	
Chronic kidney disease	0 (0-0)	0 (0-0)	0 (0-0)	0 (0-0)	.72
Coronary artery vasculopathy	0 (0-0)	0 (0-0)	0 (0-0)	0 (0-0)	.57
Acute cellular rejection	0 (0-2)	0 (0-2)	0 (0-0)	0 (0-2)	.39
Antibody mediated rejection	0 (0-1)	0 (0-1)	0 (0-0)	0 (0-1)	.53
Infection	0 (0-1)	0 (0-1)	0 (0-0)	0 (0-1)	.71
PTLD	0 (0-0)	0 (0-0)	0 (0-0)	0 (0-0)	.55

Abbreviation: PTLD, posttransplant lymphoproliferative disease.

### Acute Rejection During First 6 Months Posttransplant

Of 153 patients in the cohort, 47 (31%) had acute rejection during the first 6 months posttransplant. There was no difference in the incidence of rejection in COI groups. Fifteen patients (30%) in the very low or low, 3 patients (18%) in the moderate, and 29 patients (34%) in the high or very high COI group had rejection during the first 6 months posttransplant.

### Freedom From Death or Retransplant

**Figure 2.**
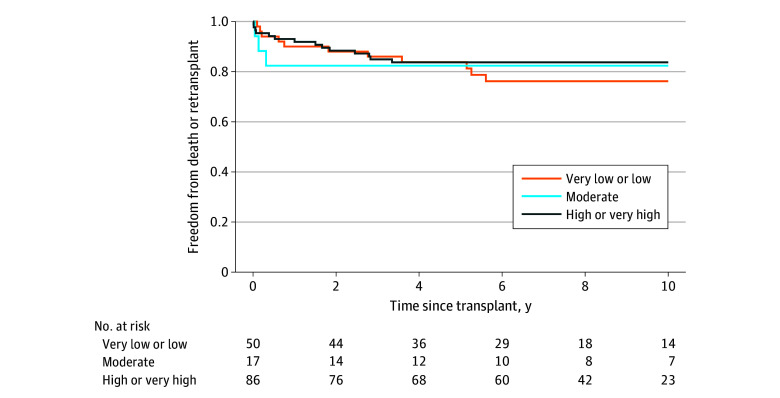
Freedom From Death or Retransplant Among Pediatric Heart Transplant Recipients by Childhood Opportunity Index Level Log-rank test comparing survival curves, *P* = .07.

Graft loss was observed in 11 patients during the first 6 months posttransplant (all deaths), 12 patients between 6 months and 3 years posttransplant (all deaths), and 5 patients after 3 years posttransplant (4 deaths and 1 retransplant). There were no differences between COI groups in risk for graft loss (Figure 2).

## Discussion

Several studies, including a study of pediatric HT recipients from 1991 to 2005 from our own center, have shown Black race and low SES to be associated with a higher risk of rejection and graft loss in pediatric HT recipients.^3,4,15,16,17^ The current study sought to evaluate the association of SES with a broader set of posttransplant outcomes in pediatric HT recipients who underwent transplant from 2006 to 2019 at our center. As expected, we found that children with low SES, defined as those living in very low or low COI neighborhoods, were more likely to be non-White. However, we found no difference in any transplant-related outcome among COI groups, including graft rejection during the first 6 months, the 3-year MATE-6 score (representing cumulative burden of 6 major posttransplant events), individual MATE components, or risk of death or graft loss during the entire follow-up period.

Our group has evaluated pediatric HT outcomes through the lens of social determinants of health since 2009.^15^ At the time, the association of Black race with rejection and graft loss across solid organs was well known; however, the emphasis within investigating potential explanation was on genetic and immunologic differences between racial groups.^18,19,20,21^ One study in pediatric HT recipients has since shown that genetic and clinical factors explain only 8% to 19% of the excess risk associated with race.^22^ Having observed that low SES patients often struggled with demands of post-HT care irrespective of race, we analyzed 135 consecutive pediatric HT recipients between 1991 and 2005 at our center. The results showed a significant association of low SES with rejection and graft loss in pediatric HT recipients after adjusting for patient factors.^15^ Since then, these observations have been confirmed in several multicenter cohorts of HT recipients, in children and in adults, including in analyses of recent-era HT recipients.^3,4,17,23^

The results of the current study are different. Not only did the current study fail to show an association of SES with chronic effects of immune suppression (infection, kidney dysfunction, or lymphoproliferative disease), the analysis did not find a difference among COI groups in risk of rejection or graft loss either. Most patients had no or mild events (Figure 1B, Table 3), and graft survival improved across SES compared with the 1991 to 2005 cohort. The potential mechanism for these results include salutary effects of clinical and quality improvement practices at our center over the years; however, the potential of a small sample size and false-negative results needs to be considered first. Using the mean (SD) MATE-6 score of 10 (4) in the original cohort (Pediatric Heart Transplant Study, HT during 2005-2014), we had estimated that our cohort had adequate power to detect a clinically meaningful difference in MATE-6 scores in the very low or low COI group compared with other groups.^5^ The mean (SD) MATE-6 score in our cohort (3.6 [6.7]) was substantially lower than in the original MATE cohort, suggesting that the contemporary care of pediatric HT recipients is associated with lower morbidity than in the original MATE cohort. Notably, the TEAMMATE trial investigators^24^ have presented a similar analysis in 181 patients with COI data (HT during 2019-2022). They found that the average MATE-6 score in patients in the very low or low COI group was twice as high as that in the high or very high COI group and 50% higher than in the moderate COI group.^24^ Although we may be unable to detect true differences of a small magnitude, the overall MATE scores among the 3 COI groups were very similar, and did not show any trend.

An alternative explanation may be a combination of systemic changes beginning around 2006 that had salutary effects on patient outcomes and disparities at our center. Coincidently, the state of Massachusetts passed a health care reform with the aim of providing health insurance to all its residents in 2006, improving overall access to health care in the state. Second, our center initiated a steroid avoidance posttransplant IS protocol for HT recipients at low risk of AMR in 2006 consisting of induction therapy for 5 days, followed by tacrolimus and mycophenolate maintenance IS, with standardized periodic monitoring of trough IS levels, and standardized infection prophylaxis.^14,25^ Immune suppression protocol for patients at higher risk of AMR was also standardized. A predischarge checklist after HT ensured that 2 primary caregivers for every patient received full transplant care education. A quality improvement initiative ensured that target levels of IS medications (which adjust with duration since HT), were documented in the clinic note at every visit. Weekly clinician team meetings have included discussion of all current inpatients as well as all outpatient visits. Annual rates of rejection, infection, and lymphoproliferative disease have been measured and analyzed for quality improvement. All these initiatives have likely contributed to improved overall outcomes. Finally, lower MATE scores in our cohort compared with the original MATE cohort may also be because half of our cohort received HTs after 2014, and HT outcomes have continued to improve everywhere over time despite higher patient complexity.^26^ It is important to note that our study cohort is not limited to low-risk patients but includes all consecutive patients during the study period. A recent multicenter study^27^ based on clinician survey acknowledged implicit preference for White and higher SES patients, and explicit preference for higher education families among pediatric HT clinicians. To the extent that such clinician preferences contribute to disparate outcomes, standardized protocol-driven care and quality improvement initiatives that affect all patients may be effective antidotes to functional effects of practitioner bias by improving overall outcomes.

Two clinical practices may have had a direct effect on narrowing disparities by benefiting low SES patients disproportionately, although they were not initiated with that intent. First, our center has provided resources for a clinical social worker to attend all outpatient clinics for HT recipients, thus addressing any psychosocial and economic concerns with urgency. Second, the team nurses maintain a red-zone file that has daily updates on patients who may be at near-term risk of an adverse event. Examples include patients with an intercurrent illness, or recent subtherapeutic IS levels who need guidance toward appropriate action until the risk abates. Finally, the first step in reducing socioeconomic disparities in health outcomes requires clinician awareness, and our earlier work demonstrating disparate outcomes among different SES groups in our patient population certainly raised collective awareness. Lack of disparities in the current study may in part be due to a Hawthorne-like effect in clinical practice, even if clinicians are not participants in an experiment.

There are important clinical implications of our results. The study cohort had excellent clinical outcomes with low MATE scores in all event categories. It is notable that the very low or low COI group was predominantly non-White, and had equivalently low MATE scores, suggesting that despite historic differences in outcomes by race, there is no medical basis for those findings. Equal access to care, increased awareness of inequities, standardized protocol-driven care, and measures directed at the most vulnerable patients in clinical practice may remedy the effects of implicit bias across socioeconomic divides and improve patient outcomes for all pediatric HT recipients.

### Limitations

This study has limitations. Being a single center study, the sample size is not large enough to detect small differences. The racial composition of the cohort reflected the center referral pattern and demographics of the northeastern US, and did not allow comparison of outcomes by race. SES disparity in pretransplant referral patterns from other centers is not measurable, and if it was present, it might have biased the results toward the null. COI was evaluated at the time of HT, and did not account for social mobility following transplant. Furthermore, although we suggest potential explanations for our findings, we have not measured the direct effect of any of these measures in this study. Finally, the results may not be generalizable to HT recipients at other centers.

## Conclusions

In this cohort study of pediatric HT recipients, there was no difference in posttransplant outcomes among recipients stratified by SES, a notable improvement from prior studies. Socioeconomic factors have been shown to be important determinants of health outcomes, including in pediatric HT recipients. Our results suggest that with awareness and universal application of high-quality patient care, it may be possible to overcome socioeconomic disparities in outcomes in patients with pediatric HT. Continued awareness and use of resources to mitigate disparities is critical in maintaining such results.

## Notes

Supplement.
Data Sharing Statement
