# Supplementary material for: Socioeconomic Status and Major Adverse Transplant Events in Pediatric Heart Transplant Recipients
Source: JAMA Netw Open. 2024 Oct 3;7(10):e2437255. doi: 10.1001/jamanetworkopen.2024.37255 (PMC11450513; doi:10.1001/jamanetworkopen.2024.37255)
Supplement: Supplement. — Data Sharing Statement [file jamanetwopen-e2437255-s001.pdf]

# Data Sharing Statement

Hartje-Dunn. Socioeconomic Status and Major Adverse Transplant Events in Pediatric Heart Transplant Recipients. *JAMA Netw Open*. Published October 03, 2024. doi:10.1001/jamanetworkopen.2024.37255

**Data**
**Data available:** No

**Additional Information**
**Explanation for why data not available:** Being from a single center, the data are identifiable even if demographic data are removed.
